# The energy conversion in active transport of ions

**DOI:** 10.1073/pnas.2116586118

**Published:** 2021-11-03

**Authors:** Signe Kjelstrup, Anders Lervik

**Affiliations:** ^a^PoreLab, Department of Chemistry, Norwegian University of Science and Technology (NTNU), 7491 Trondheim, Norway

Since their discovery in the middle of the last century (1), with the subsequent Nobel prize to Skou in 1997, the family of biological ion pumps (2–4) has posed a challenge to the scientific community at large. The pumps utilize the Gibbs energy of hydrolysis of adenosine triphosphate (ATP) to move ions uphill across a membrane. How do they function? A much-studied case is Ca-ATPase (adenosine triphosphatase), which acts to control the level of the Ca${}^{2+}$ content in muscle (Fig. 1 has a schematic representation). When inactive, the pump forms a closed gate in the lipid membrane. The gate opens to the internal side when ATP is supplied to the external side of the membrane. The computational results of Kobayashi et al. (5) in PNAS provide support for several critical events. First is the release of calcium ions during opening, following right after a structural change in Ca-ATPase. Next is the rapid protonation of side chains that prevents rebinding of Ca${}^{2+}$, combined with interactions between lipid molecules and transmembrane helices that help stabilize the structure. Using rare event molecular dynamics simulations, Kobayashi et al. (5) captured the structural changes in great detail and obtained Gibbs energy profiles that demonstrate the importance of a rapid exchange between Ca${}^{2+}$ ions and protons upon the release of Ca${}^{2+}$.

**Fig. 1:**
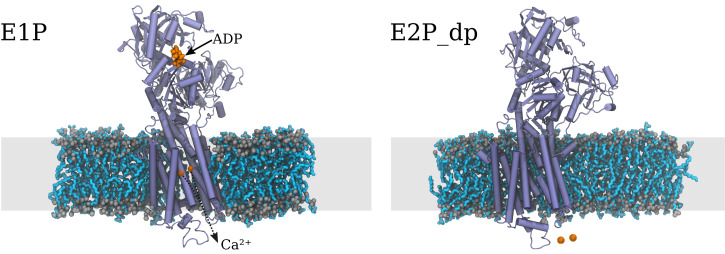
Schematic representation of two of the enzyme states studied by Kobayashi et al. (5): the adenosine diphosphate (ADP)–sensitive phosphoenzyme state, “E1P,” and the low-energy phosphoenzyme state, ”E2p_dp.” The structures were obtained from the supporting information in ref. 5. Here, E1P denotes the state of the enzyme where ADP and Ca${}^{2+}$ ions (both shown in orange) are bound to it. The ions are bound deeper within the transmembrane helices but are here depicted in front for clarity. E2p_dp denotes the state of the enzyme where the Ca${}^{2+}$ ions are released to the lumen side of the membrane. The E2P_dp state has the same protonation state as E1P. The enzyme undergoes large structural changes in the transition from the E1P state to the E2p_dp state, and a funnel-like path is opened, widening from the location of the ions inside the enzyme to the lumen side.

The transport of ions by the enzyme has been observed and described in great detail in the past by other groups (6–8). The stoichiometry of the pump and its relation to structural changes are known with astounding detail (9, 10).

The transitions between different states are challenging to investigate by all-atom simulations since the timescale for these transitions (milliseconds or slower) is orders of magnitude larger than achievable by standard brute force atomistic simulations. This does not mean that the actual transition itself is a slow process. In a brute force simulation, most of the computational time is spent exploring the stable state, before the conditions are “just right” to trigger the transition. After initiated, the transition proceeds rapidly. In their work, Kobayashi et al. (5) circumvented this timescale problem by focusing directly on the fast, but rare, transition events. Consequently, the authors could use short-timescale simulations to obtain information about the transition event.

Nevertheless, the theoretical problem still exists. How do we create a physical–chemical formulation of the molecular mechanism, which involves transforming the scalar chemical energy into a vectorial mass transport on the macroscale? Additionally, can such a description also include the well-documented heat production that follows from the operation of Ca-ATPase (11–14)? The inclusion of such heating effects would allow us to quantify contributions to nonshivering thermogenesis.

Answers to these questions are needed to fully understand the pump operation and to provide a physical–chemical description of this operation. While the pump efficiency (6, 15) can be computed from black box considerations, a local description is needed to capture molecular events and dissipation during energy conversion. Such a description must localize the energy that is dissipated as heat. A fully general physical–chemical description of the pump function must, therefore, include the temperature as a variable, in addition to a precise formulation of the ion transport and the pump slippage. Only then will we be able to link the local physical–chemical processes to supercellular events.

The findings of Kobayashi et al. (5) in PNAS for Ca-ATPase are very well suited to bring the discussions of these points to the next level, as we shall comment on below.

## The Free Energy Landscape with Six Transition States

Kobayashi et al. (5) presented Gibbs (or free) energy profiles along a path that takes the enzyme from one transition state to another (figure 4E in ref. 5). Knowledge of this energy landscape sets the stage for theoretical considerations. We can measure the degree of advancement along the path in the Gibbs energy landscape by a collective variable *γ*. In the work of Kobayashi et al. (5), the collective variable was based on the coordinates of the C*α* atoms and carbon atoms of selected side chains in the protein. In previous theoretical work (16), we have taken this coordinate to represent the advancement of the ATP hydrolysis. The Gibbs energy profile of the reacting complex (i.e., the reactants and products in a broad sense) can then be denoted μ(γ), where *μ* is the Gibbs energy of the reacting mixture at any moment in time. The six transition states then correspond to six particular values of *γ*, and paths in the *γ*-space describe transitions. A point on a path between states represents an intermediate state (there is no need for a complete conversion into products).

With a single collective variable, “shortcuts” between states (i.e., direct connections between two states that are separated by one or more intermediate states) cannot easily be described. Adding such shortcuts would allow theoretical modeling of cases with variable stoichiometry, such as thermogenesis (17). For instance, one could include as an additional collective variable *γ_d_*, representing the degree of advancement of the diffusing ion in the state space covered by coordinates $\left(\gamma ,\gamma_{d}\right)$. The Gibbs energy landscape will then be a three-dimensional surface in a two-dimensional (2D) collective variable space. In the future, the approach of Kobayashi et al. (5) could be used to explore a larger-energy landscape and then, perhaps give more information about additional functions of the enzyme.

While the above are wishes for the future, the Gibbs energy profiles by Kobayashi et al. (5) can already be used to approximate activation barriers. It is, therefore, next at hand to ask for kinetic information on the processes. In this context, Hill’s cycle diagrams (18) [leading to the familiar Post–Albers scheme (19)] are an option for a theoretical framework. Here, we shall rather point at another theoretical opportunity in order to be able to address the issue of coupling between driving forces and possible heating effects.

## Enthalpic and Entropic Barriers

Consider first some overall conditions for the pump in action. The reaction Gibbs energy of ATP hydrolysis, $\Delta G_{\text{ATP/ADP}}$, provides a value for the ideal (maximum) work that can be performed by the pump. The negative Gibbs energy is around 50 kJ/(mol ATP converted) at physiological conditions (6, 20). This is now equal to the ideal work, $W_{\text{ideal}}$, obtainable for a fully reversible pump operation. In reality, conditions are not reversible, so the value will be divided between the actual work done, *W*, and the energy dissipated as heat at temperature *T*, Tσ:

(1) $$-\Delta G_{\text{ATP/ADP}}=W_{\text{ideal}}=W+T\sigma .$$

Here, *σ* is the entropy production, which also will determine the relevant driving forces acting during pump operation (17). The dissipation Tσ is a function of the rates of the processes involved.

A first overview of the situation is given by Eq. **1**. The term Tσ reflects the irreversibility or the frictional heat of the processes involved. This term is always positive, no matter which direction the processes take, so $W<W_{\text{ideal}}$. How can we envision the creation of the work, *W*?

It is known that the entropy change in the ATP hydrolysis is small compared with the enthalpy change of the reaction (13). The simulations of Kobayashi et al. (5) document that the hydrolysis leads to large structural changes in the enzyme. This structural change is compatible with a conversion of the reaction Gibbs energy into potential energy. Such an explanation also fits well with the observation that the pump can be reversed (21).

The uphill transport of Ca${}^{2+}$ has a corresponding Gibbs energy change. Among the two contributions to this, the enthalpic contribution is probably smaller than the entropic one. Kobayashi et al. (5) discuss the feature that the entropy profile of the energy conversion in the pump is not taken into account. When we want to describe the pump function, this may become essential (22). The thermodynamic driving force for the conversion of a scalar to a vectorial process must be sought in the derivative −dμ/dγ. After the reaction Gibbs energy has been stored as potential energy in the new structure, the variation in *μ* will predominantly depend on the entropy variation of the ion exchange along the path. The structural step and the ion translocation are major parts of the total *W*.

Still, there is the pressing question of how to understand and deal with heat production. Evidently, the entropy production Tσ is not sufficient, as it cannot describe the reversible heat effects that are observed.

Reversible processes can also be associated with heat transport, including at isothermal conditions. This happens, for instance, in a Carnot cycle. Here, the reversible pump operation is associated with a reversible heat transfer to the cytosol, a fact that has been used to explain nonshivering thermogenesis. Such a reversible heat production has its origin in transported entropy or fluxes that constitute *σ*. The effect is similar to the Peltier effect in electrochemistry. It will change sign upon a change in the process direction. In the present case, the process of ion exchange is probably dominated by the entropy change that follows the ion exchange since there are negligible binding energies for the ions in the cytosol or lumen. This entropy change is large and can also vary largely.

## Coupling of Fluxes in State Space

The early descriptions of active transport across membranes dealt with the membrane as a black box and used a discrete formulation [for instance, the classical books of Katchalsky and Curran (23) and Caplan and Essig (24)]. The energy conversion from the scalar reaction Gibbs energy into the directed transmembrane gradient in the chemical potential of the ion was assigned to a vectorial coefficient. With the idea of symmetry breaking at the interface (25), the problem of the vectorial coefficient disappears. Coupling is instead described by the interaction of scalar components of the fluxes perpendicular to the membrane interface. With the advent of mesoscopic nonequilibrium thermodynamics (26), the black box becomes obsolete. A continuous path along a *γ*-coordinate can then be assigned to the active transport problem. Flux-force relationships are, therefore, also no longer necessarily linear, another problem that has hampered theoretical progress. Their nonlinear relationships can be understood as stemming from an integration over *γ* space. As discussed above, it is a path in *γ* space that has now been made visible by Kobayashi et al. (5).

Central for the development of mesoscopic nonequilibrium thermodynamics is the assumption of local equilibrium along such a path. Clearly, with the results of well-defined transition states, approachable as crystals, it is reasonable that this assumption holds. We are then justified to define the entropy production in the *γ* space. This is also important for the possibility to define a local temperature and find it from atoms’ kinetic degrees of freedom.

For the ion pump, there are three independent flux-force terms: due to 1) the chemical reaction, 2) the ion exchange, and 3) the heat transport (16, 17). The three flux-force products define the magnitude of the frictional losses in Tσ. However, through coupling (in particular, between the heat flux and mass flux), we can describe the reversible component of the heat flux in terms of an entropy flux and explain the heating of the cytosol during pumping (21). After the entropy production of the membrane is found, the energy efficiency can be related to slippage. Also, more importantly, a dynamic model can be proposed for further experimental tests and reduction of data.

## Conclusion and Perspectives

This commentary on the work of Kobayashi et al. (5) points out possibilities that their achievement has produced. In their simulations, they have obtained a detailed picture of structural changes that take place during the operation of Ca-ATPase. They have also obtained a one-dimensional Gibbs energy profile, characterized by the degree of advancement between states. We see this as a basis for future expansions into more complex energy landscapes, which will advance our understanding of the pump operation. For instance, as discussed above, expanding the description into 2D may help us include heating effects and describe the pump as the slipping, heat-generating engine it is, according to observations.

Biological ion pumps are usually studied under isothermal conditions, and much is known about their operation in these conditions. The convincing results of de Meis and others (11, 12, 14, 21) on Ca-ATPase need, however, to be included into current descriptions of the isothermal action. We propose that the ion exchange associated with the pump leads to an entropy change and that this can be used to explain nonshivering thermogenesis. Like in batteries, the entropy change due to a reaction has a thermal signature (the entropy of reaction times the temperature). We may thus speculate that ion pumps can be viewed as heat pumps. A theoretical description, now lacking to a large degree, may help distinguish between mechanisms of heat production of a reversible or irreversible nature.

The work of Kobayashi et al. (5) provides opportunities for experimentalists, theoreticians, and simulators; their detailed picture advances our current understanding and points toward the next level.
